# Using Information and Communication Technology in Home Care for Communication between Patients, Family Members, and Healthcare Professionals: A Systematic Review

**DOI:** 10.1155/2013/461829

**Published:** 2013-04-10

**Authors:** Birgitta Lindberg, Carina Nilsson, Daniel Zotterman, Siv Söderberg, Lisa Skär

**Affiliations:** Division of Nursing, Department of Health Science, Luleå University of Technology, 971 87 Luleå, Sweden

## Abstract

*Introduction.* Information and communication technology (ICT) are becoming a natural part in healthcare both for delivering and giving accessibility to healthcare for people with chronic illness living at home. *Aim.* The aim was to review existing studies describing the use of ICT in home care for communication between patients, family members, and healthcare professionals. *Methods.* A review of studies was conducted that identified 1,276 studies. A selection process and quality appraisal were conducted, which finally resulted in 107 studies. *Results.* The general results offer an overview of characteristics of studies describing the use of ICT applications in home care and are summarized in areas including study approach, quality appraisal, publications data, terminology used for defining the technology, and disease diagnosis. The specific results describe how communication with ICT was performed in home care and the benefits and drawbacks with the use of ICT. Results were predominated by positive responses in the use of ICT. *Conclusion.* The use of ICT applications in home care is an expanding research area, with a variety of ICT tools used that could increase accessibility to home care. Using ICT can lead to people living with chronic illnesses gaining control of their illness that promotes self-care.

## 1. Introduction

Due to an ageing population and a shortage of hospital beds, it has become a challenge to find new ways to support and care for people with chronic illness living at home. Living with chronic illness changes the lives of those affected, who are often in need of support and nursing care in their homes [1–3]. eHealth has the potential to become a means of providing good care at home [4], which is especially challenging with regard to this emerging field [5]. eHealth refers to information and communication technology (ICT) tools and services for health, whether the tools are used behind the scenes by healthcare professionals or directly by patients and their relatives [6]. ICT tools can be used to access a wide variety of technological solutions for communication, including text messaging, gathering and monitoring data, diagnosis and treatment at distances, and retrieving electronic health records [5, 7]. According to the World Health Organization (WHO) [8], eHealth is used in the healthcare for transmission of digital data, including data stored and retrieved electronically to support healthcare, both at the local site and at a distance. 

E-Health includes the interaction between patients and health service providers or peer-to-peer communication between patients and/or health professionals. Interest has primarily focused on the use of ICT tools in the care of older [9] and severely chronically ill people [10]. Although ICT has been increasingly used in healthcare in recent years, efforts across countries have been fragmented and could benefit from improved cross-border coordination. eHealth tools and services have been widely introduced and implemented, and the potential benefits ICT can bring people with chronic illness will increase significantly [6].

## 2. Aim

The aim was to review existing studies describing the use of ICT in home care for communication between patients, family members, and healthcare professionals. 

The particular objectives of the review were the following:

- to provide an overview of characteristics of studies describing the use of ICT in home care,
- to describe how ICT was used for communication in home care,
- to describe the benefits and drawbacks of the use of ICT in home care.

## 3. Method

The design for conducting this systematic review was guided by DiCenso et al. [11], with the following steps taken: for formulating a research question, conducting a literature search, applying inclusion and exclusion criteria, abstracting data, and undertaking an analysis.

### 3.1. Selection Criteria

The inclusion criteria for this literature review were set as follows: (1) ICT interventions; (2) communication between any healthcare professionals, patients, and/or family members; (3) studies published in scientific journals; (4) studies published between 2000 and 2010; and (5) in the English language. Criteria for exclusion were ICT interventions that included technological systems not involving people (no active patient acceptance) such as monitoring by camera, alarm systems, and use of ordinary telephones, noting that telephones can be used complementarily to other techniques. Letters, editorials, and news items were also excluded.

### 3.2. Search Strategy

In the literature search the following electronic bibliographic databases were used: PubMed, Scopus, and CINAHL. Search limits were set to English language studies published in scientific journals from 2000 to June 2010. The search terms and search strategy were customized for each database to search completely and exactly. The search strategy included thesaurus terms (MeSH terms and subject headings) combined with free-text words. Examples of main search terms used were telemedicine, information and communication, ICT, technology, e-health, home care, home, and nursing. To maximize the search results, multiple sets of search terms were used. The search was done until an overlap in the studies was observed. All studies retrieved from the search in databases were imported into a reference manager (EndNote). The literature searches resulted in 1,276 studies; after duplicates were discarded by EndNote, 923 studies remained. A search alert was created to get the latest published studies, which resulted in 11 additional studies. The final total to be reviewed was 934. The literature search was performed with support from librarians. 

### 3.3. Selection Process

**Figure 1:**
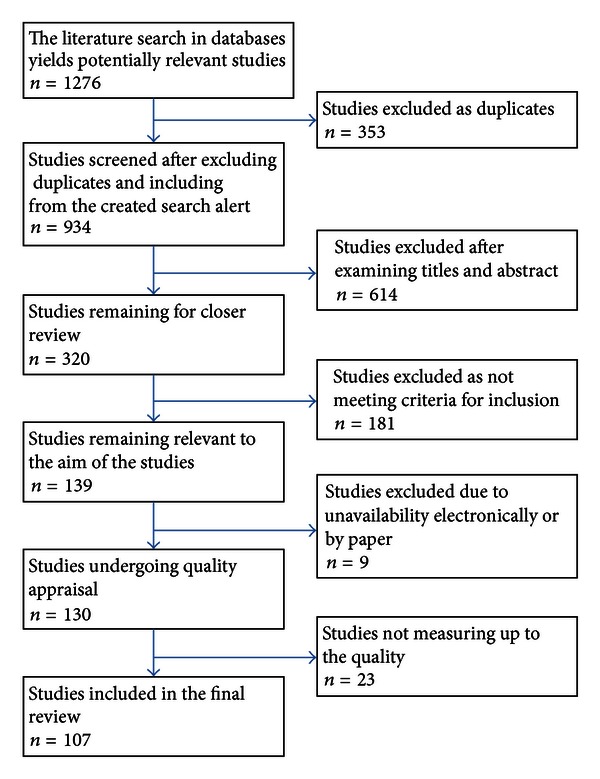
Flow chart of search result.

A first selection was based on titles and abstracts of the 934 studies to identify whether or not they were within the scope of the research question. Next, a selection based on inclusion criteria was conducted, with focus on studies of ICT applications used in home care. After this selection, a total of 320 studies remained for closer review. The full-text version of the studies was then read and initially categorized based on type of communication applied in the studies. Two authors read all the studies independently. To increase reliability they discussed ambiguities of inclusion criteria until consensus was reached. This reduced the number to 139 studies relevant to the research question. However, nine relevant studies were unavailable both electronically and in paper form, which thereby were excluded from this study, leaving 130 studies. The selection process for the studies reviewed is presented in Figure 1. 

### 3.4. Quality Appraisal

All eligible studies (*n* = 130) were evaluated for scientific quality on a three-grade scale: high scientific quality, good scientific quality, and fair scientific quality. The grading system is used by The Swedish Council on Technology Assessment in Health Care (SBU) for systematic reviews [12–14]. The quality appraisal was performed in accordance with a previously presented method for quality appraisal [15–18], which was chosen to be appropriate. In appraising the scientific quality of each study, protocols were used to extract data. Different protocols were used for studies with a quantitative approach and for studies with a qualitative approach. In the protocol for quantitative studies the items focused mainly on exclusion, sample procedures, intervention, dropouts, randomization, similarity of groups, blinding, outcomes, statistical procedures, ethical considerations, validity and reliability of instruments used, and possibility of generalization of results. In the protocol for qualitative studies the items focused mainly on context, ethical reasoning, procedure of sample, data collection, analysis procedures, saturation, clarity and logic of results, theoretical framework, theory generation, and description of main results. The protocols contained questions to be answered with yes/no/unclear and additional space to comment on the relevance of each item and for the extracted data. The number of questions answered yes was divided by the total number of questions and thereafter converted to percentage. Willman et al. [15] state that the use of percentage makes it possible to weight and compare different study's methodologies. As recommended [15] the percentage was transformed to high scientific quality (80–100%), good scientific quality (70–79%), and fair scientific quality (60–69%). The studies that scored less than fair were excluded (*n* = 23), as they were considered not to be of sufficient scientific quality to be included. The quality appraisal was performed by two of the authors, initially together to obtain an equal assessment, but thereafter independently. When uncertainties arose, the authors discussed the result of the quality appraisal until consensus emerged. After the quality appraisal was undertaken, 107 studies remained. 

### 3.5. Data Abstraction

The remaining 107 studies were classified as relevant to the research question and met the inclusion and quality criteria for being included in the data abstraction. A list of all included studies can be found in Table 6. Each of the included studies was given an indexation and then categorized according to a number of different areas based on the following characteristics: country of origin, year of publication, study approach, journal, communication strategies, type of technology, type of communication, disease diagnosis, and quality appraisal. Thereafter, data from each of the included studies were extracted and entered into a matrix. 

## 4. Results

The result presentation is divided in two parts; general and specific results.

### 4.1. General Results

The general results give an overview of characteristics of studies describing the use of ICT applications in home care. The results are summarized in areas including study approach, quality appraisal, publications data, terminology used for defining the technology, and disease diagnosis.

#### 4.1.1. Studies' Approach

Most of the included studies had a quantitative approach. Only about one-fifth had a qualitative approach. Further, some of the studies used mixed methods, with both qualitative and quantitative approaches (Table 1). Twenty-one studies were part of larger projects. 

#### 4.1.2. Quality Appraisal

In the critical quality appraisal of all 107 studies, just under half were rated as high scientific quality (*n* = 48). That number was compared to studies rated as good scientific quality (*n* = 23) and fair to good scientific quality (*n* = 36) (Table 1). When comparing the quality appraisal between qualitative and quantitative approaches, differences could be noted. A greater proportion of the qualitative studies were rated as high scientific quality. In comparison, less than half of the quantitative studies were rated as high scientific quality. The opposite was the case with qualitative and quantitative studies rated as fair scientific quality. Good scientific quality ratings were found in both qualitative and quantitative studies. 

#### 4.1.3. Publication Data

**Figure 2:**
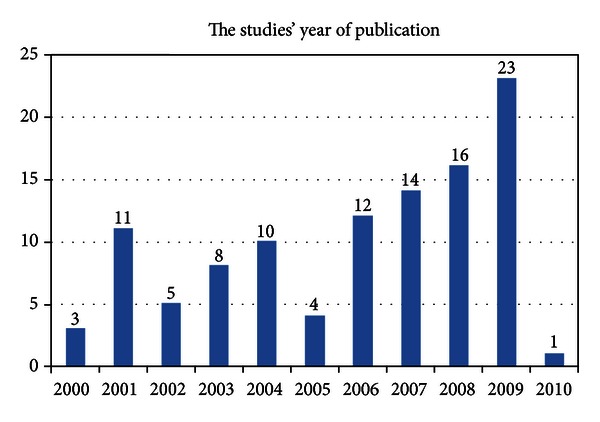
Number of studies published per year between 2000 and June 2010.

All of the 107 included studies were published between January 2000 and June 2010, so only part of year 2010 was included. During this period the number of publications increased by time, with about half of the included studies (*n* = 53) published between 2007 and 2009. Note that 2009 alone represents 23 studies of the total publications (Figure 2). 

The studies included were published in 69 different scientific journals. The two most common journals were Journal of Telemedicine and Telecare (*n* = 15) and Telemedicine Journal and e-Health (*n* = 12), together representing almost one-quarter of the total number of studies. The rest of the studies (*n* = 80) were spread over a variety of other journals (*n* = 67). The impact factor in the journals ranged between 0.348 and 14,293.

The majority of the studies were performed in North America (*n* = 67). About one-third of the studies were done in Europe (*n* = 34), with United Kingdom, Sweden, and Italy being the most prominent. Only a few studies (*n* = 6) were conducted outside North America and Europe; those were done in Asia (*n* = 5) and Australia (*n* = 1). Three studies were carried out in cooperation between different countries, but only one study was a combined study involving the continents of North America and Europe (Table 2).

#### 4.1.4. Terminology Used for Defining the Technology

The results show that 13 different terms were used to define the technology utilized to increase accessibility to home care services and home nursing. The most frequently used terms were telehealth, telemedicine, technology, and telecare. Telehealth and telemedicine together (*n* = 59) account for more than half of the terms used in the included studies. Other terms used three times or more were e-Health, ICT/IT, telehealthcare, telemonitoring, and telenursing. Further, in some studies other terms were used as follows: e-rehabilitation, teleassistance, and telerehabilitation (Table 3).

#### 4.1.5. Disease Diagnosis

**Figure 3:**
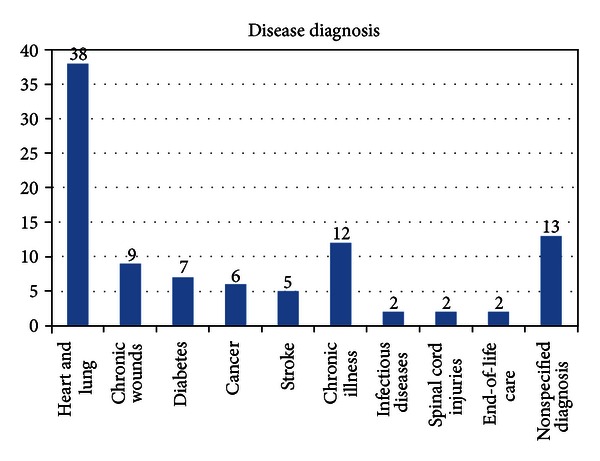
Number of studies per disease diagnosis.

The ICT applications were used in healthcare for a wide range of different conditions through the life span. In the majority of the studies (*n* = 86), the technology was developed specifically for supporting people with chronicle illness living at home. The most frequent diseases studied were heart and lung diseases, chronic wounds, diabetes, cancer, and stroke. Chronic illness was used in 12 studies without any definition of the specific disease. Other conditions were, for example, infectious diseases, spinal cord injuries, and end-of-life care. A number of studies included did not specify the diagnoses (Figure 3). 

### 4.2. Specific Results

The specific results describe how ICT was used for communication in home care and benefits and drawbacks within the use of ICT in home care. The results are summarized in the following main areas: type of technology, communications between participants, and benefits and drawbacks of the use of ICT.

#### 4.2.1. Types of Technology

Three fields of applications were found to be prominent in the use of ICT in homecare: video technology, text messages and health monitoring. An important result was that a mix of more than one ICT applications was used in several studies (*n* = 31). A small number of studies included all types of ICT applications above. In some of the studies, a mix of text and pictures and/or audio was used. In a few studies digital images were used. Some studies did not specify the used ICT application (Table 4).

*Video Technology.* The most frequently used type of technology was video technology (*n* = 53); the number includes studies using more than one ICT application. In several of those studies (*n* = 31), the main focus of the intervention was the use of videophones or videoconferencing. Another use of video technology was to complement patient health monitoring (*n* = 22). It is notable that web-based video conferencing was used only in a small number of studies (*n* = 3). In all studies involving parents of children with chronicle illness, video technology was used to communicate. 

Video technology was used with different types of applications. Examples of use were guiding patients in their use of medical equipment and to improve self-management, via video-based home telecare services. Another use was teleadvice given by clinical nurse specialists in different areas to community nurses. Videoconferencing was used between patients/family members and healthcare personnel for education and psychosocial or emotional support. Another way to use videoconferencing was to enable interactions between patients and nurses. Consultation via videoconferencing in the patient's home was used instead of visits to the hospital, which enabled access to experts to a greater extent. Virtual nurse visits after, for example, discharge from the hospital, were offered to both patients and family members.

*Text Messages.* As shown in many studies (*n* = 30), a common way of communicating was via text messages. For sending text messages, websites or web-based programs were used in some studies (*n* = 10). Handheld platforms, such as mobile phones, laptop computers, or text telephones, were used by patients to both send and receive information as well as to communicate (*n* = 12). In other studies (*n* = 8), mobile phones or hand held equipment was used to send text messages.

For example, text messages were used for sending messages to patients with self-care advice as a response to symptoms and test results they had reported. Another way to use text messages was by electronic diary for home monitoring to improve communication between patients and healthcare professionals. An electronic messaging programme via computers and mobile phones or e-mail and video mail messages was used, enabling nurses and patients to exchange messages to and from anywhere. Via a symptom management system, patients can receive messages in their daily management of symptoms.

*Health Monitoring.* About half of the total studies (*n* = 52) included health monitoring, focusing on patients who sent health data to be analyzed by healthcare professionals. In most of the studies that looked at monitoring patient health, text messaging or video technology was used to communicate the data (*n* = 35). Other forms of communication were also used, including the telephone (*n* = 17). Health Buddy, was the most commonly used device for monitoring patient health (*n* = 8). Health Buddy, a system that connects patients in their homes with care providers, is a telehealth device that collects and transmits disease management information about a patient's condition including vital signs, symptoms, and behaviors. Types of patient health data collected from health monitoring systems in real time were, for example, weight, blood pressure, heart rate, and pulse. 

#### 4.2.2. Communication between Participants

Different types of communication via ICT were described as being used between participants, who were typically nurses, healthcare professionals, patients, or family members. The most frequent line of communication in the studies was between patients and nurses or other healthcare professionals. ICT was used most for communication between nurses and patients. In 24 studies, the patient was not the focus for communication. Instead, it was common for the technology to be used for communication with family members. In five of the studies with a focus on family members, the ICT was developed for healthcare personnel giving support to parents. In some studies, the communication was merely between healthcare professionals and neither patients nor family members were part of the communication. The review shows that people living with illnesses at home and healthcare professionals gave positive responses from using different ICT applications for healthcare in communication with each other (Table 5).

### 4.3. Benefits and Drawbacks with the Use of ICT in Home Care

Results of the included studies were predominated by positive responses from the use of different ICT applications in home care from both people living with chronic illnesses and healthcare professionals. For example, healthcare professionals' opinions were that their work was facilitated. Most studies show that communication between healthcare professionals and patients living at home was improved by using various ICT applications, as improvement in management of symptoms in daily life. It was revealed that various ICT applications can be advantageous to use in follow-up care of patients at home. Another benefit of using ICT applications in home care was found to be an improved accessibility. Results from studies show that using ICT in communication in home care can be cost saving but also the opposite. However, the use of ICT cannot replace a face-to-face encounter but can be used as a complement.

## 5. Discussion

The aim of this study was to review existing studies describing the use of ICT in home care for communication between patients, family members, and healthcare professionals. This review provides an overview of characteristics of studies describing the use of ICT applications in home care. The results show that ICT in home care is an expanding field of interest, with a variety of ICT tools beginning to be evaluated significantly. Half of the included studies reviewed represent the year between 2007 and 2009. This may reflect the increased use of the Internet and ICT tools for care management with involvement of patients and family members' participation in care processes. Previous research [19] stated that focus has emerged from being technology focused to taking the users', that is, the patient, family members, and healthcare professionals, perspective into account. 

The review shows a trend that most studies were accomplished in North America and Europe, where the United Kingdom, Sweden, and Italy were most prominent. This is noticeable since Italy is one of the European countries in which less than 30 percent of the population uses the Internet on a daily basis. The maturity of the Internet use in daily life is an indicator of how far the digitalization of the healthcare sector should have come [19]. For instance, despite Sweden being a small country, seven of the studies included in this review were performed there, which might be explained by the fact that 75 percent of the population uses the Internet on a daily basis. 

This review shows that a wide variety of terms were used in the reviewed studies to define ICT. Most frequently used definitions were telehealth and telemedicine. This is in line with Koch's [7] review of the current state and future trends in home telehealth. The term telehealth has been broadly defined as the use of telecommunication and information technologies for provision of healthcare to individuals at a geographical distance [20]. Telehealth involves a wide variety of specific modalities including telephone-based interactions, Internet-based information, still and live imaging, personal digital assistants, and interactive audio-video communication or television [21]. Furthermore, eHealth is described as the overall umbrella field that includes both ICT and telehealth, combining use of electronic communication and information technology in healthcare [22]. This may explain the results of this review with many different terms used to define the technology.

This review describes how ICT was used for communication in home care, and an interesting result found was that the most frequent type of communication was between patients and healthcare professionals. This indicates that user focus needs to be shifting from tools for professionals to tools for patients and family members. This is in accordance with Koch [7], describing trends toward tools and services not only for professionals, but also for patients and citizens. However from a nursing perspective, there is a lack of knowledge about how to use ICT solutions to meet the needs of people with chronic illness. In specific, by performing qualitative studies people's needs related to living with chronic illness can be elucidated. A challenge in home care will therefore be to use existing ICT tools to meet caring needs of people with chronic illness based on their experiences [23]. From a caring perspective, it is important to understand ICTs impact on quality of life, quality of care, and medical impact of measureable parameters [24].

This review describes benefits and drawbacks when ICT was used for communication in home care. A variety of ICT applications are described in the review. Bardram et al. [23] stated that ICT applications used in home care must take into consideration the role technology should play in the use of patient and healthcare professionals. Neglecting this aspect may lead to technology that not provide the needed support for communication. According to Koch et al. [25], research and practice of health-enabling and ambient-assistive technologies may significantly contribute to that technical solutions are explored in a social context and in relation to individual needs. Telehealth systems in the form of online and mobile tools are already opening up the possibilities for reduced hospitalization and an increased home care [26]. Various ICT applications will thereby offer healthcare professionals to become more flexible and able to address the differing needs of individual patients [27], that is, a more person-centred care. 

The results of this review show that people living with chronic illnesses and healthcare professionals were positive to the use of ICT applications, despite that ICT cannot replace a face-to-face encounter but can be used as a complement. Across the literature, outcomes for telehealth-based services are generally comparable to outcomes for services delivered face to face [21]. According to Charlton et al. [133], the style and type of communication the healthcare professional uses influence care outcomes. A literature review [134] shows that patients with possibilities of being cared for and using telecare at home preferred a combination of telecare and traditional healthcare delivery. Therefore, ICT applications must be used as an adjunct and not as replacements for standard care; otherwise, the positive results might not be replicated [135]. Many patients prefer being involved and participating in decision making regarding the care they will receive. Despite this, caring programs will be developed without caregiver's participation [136]. 

### 5.1. Methodological Considerations

The strength of this review is the broad literature search that finally resulted in 107 studies. The literature search was systematically conducted using selected databases based on relevant search terms. Even though the database search was done with assistance from a librarian expert in that field, it is possible that some study might have been missed. To get the latest published studies, a search alert was created. A limitation of this review may be that relevant studies might have been missed because of the selection of the English language. During the selection process, a quality appraisal was conducted; thereby, the scientific quality of the included studies could be ensured. The studies included have a great variation in study designs. Therefore, it is not possible to integrate the results and give a more specific summary in this review. However, this was not the intention as the aim was broad; we wanted to find numerous studies for being able to present the state of the art in this field of research.

## 6. Conclusion

The use of ICT applications in home care is an expanding research area, with a variety of ICT applications used to increase access to home care. The result shows that ICT in home care is mostly used as a tool for communication between healthcare professionals and patients or family members. Healthcare professionals can, based on this result, advantageously use ICT applications in home care as a tool to support people living with chronic illnesses gaining control of their illness that promotes self-care. However, a great number of the included studies were performed as pilot studies. For being able to evaluate the effects of ICT applications in home care, more extensive longitudinal studies are needed. To understand more about how ICT can be adjusted to home care, multidisciplinary and qualitative studies are needed from the perspective of the patient and their close relatives.

## Figures and Tables

**Table 1:** Sample data representation.

Study quality	Study method (number of studies)		
	Qualitative (21)	Quantitative (74)	Mixed (12)
High scientific quality	15 (71%)	29 (39%)	4 (33%)
Good scientific quality	4 (19%)	17 (23%)	2 (17%)
Fair scientific quality	2 (10%)	28 (38%)	6 (50%)

**Table 2:** Number of studies per country.

Country	Number of studies
USA	62
UK	12
Sweden	7
Italy	5
Canada	4
China	2
Japan	2
Australia	1
Austria	1
Belgium	1
Denmark	1
Finland	1
Germany	1
Netherlands	1
Norway	1
Poland	1
South Korea	1
Denmark/Norway	1
UK/Germany/Netherlands	1
USA/Netherlands	1

Total of studies	107

**Table 3:** Number of studies per terminology.

Terminology	Number of studies
Telehealth	32
Telemedicine	27
Technology	11
Telecare	10
ICT/IT	7
Telemonitoring	6
Telenursing	4
e-Health	3
Telehealthcare	3
Telerehabilitation	2
e-rehabilitation	1
Teleassistance	1

Total of studies	107

**Table 4:** Overview of ICT applications used in homecare.

Number of studies (main focus for the study)	Fields of application*		
	Video technology (*n* = 53)**	Text messages (*n* = 30)**	Health monitoring (*n* = 52)**
Type of technology	49	26	17
All types	4		
Mix of text and picture and/or audio***			6
Digital images			3
Not specified type of technology	2		

Total of studies	107		

*Type of technology is divided into three fields of application (most prominent in the included studies).

**Total number of studies including this type of technology. The number includes studies using more than one type of technology.

***Included in health monitoring.

**Table 5:** Communication between participants.

Communication	Number of studies
Patient-nurse	49
Patient-other healthcare professionals	34
Family members-healthcare professionals	14
Between healthcare professionals	10

Total of studies	107

**Table 6:** Overview of studies (*n* = 107) included, interventions, and main results.

Studies	Intervention	Main results
Agrell et al. [28]	Chronically ill patients could use medical equipment in their homes guided by nurses via video-based home telecare services.	Participants were either very satisfied or somewhat satisfied with services they had received. All except one were willing to receive home telecare services in the future. The presence of telecare equipment in the home implied 24-hour-a-day access to a nurse. Some of the participants felt uncomfortable disclosing intimate information during televisits and others lamented the reduced amount of time nurses spent “socializing” as compared to in-person visits.

Ameen et al. [29]	Patients in an experimental and control group had their ulcers photographed before and after the intervention. During the intervention period, an experienced clinical nurse specialist in tissue viability gave expert teleadvice to the community nurse.	Statistically significant improvements were observed for the experimental group in the areas of dressings and management. Teleadvice can be of great benefit to community nurses in enhancing their knowledge in the practice of leg ulcer care. Provision of teleadvice can significantly improve nurses' knowledge in the care of leg ulcers. Teleadvice has implications for more efficient use of human resources and cost effectiveness in wound care.

Arnaert and Delesie [30]	Real-time interpersonal communication was used between elderly patients and nurses. Tele-nurses delivered psychosocial support and educational interventions based on three principles: contact and communication, safety and protection, and care mediation.	Telecare is an alternative care model that could be integrated into existing homecare services to provide older people with integrated health services.

Arnaert et al. [31]	Video telephones (videophones) were used for exploring attitudes of older adults with depressive symptoms in their homes.	Participants' preattitudes were dependent on their active or passive role in the learning process of the new technology. Their postattitudes were classified as ambivalent or positive. Two participants who had a positive attitude toward the videophones expressed a positive behavior use.

Artinian et al. [32]	Patients participated in nurse-managed home tele-monitoring plus usual care or in nurse-managed, community-based monitoring plus usual care. Each week during the study period, patients received telephone counseling about lifestyle modifications. A specially trained registered nurse delivered the interventions. Each participant received an electronic home BPLink monitor and BP monitoring services. The BPLink monitor is a system that enables persons to monitor blood pressure and heart rate at home and send readings to the investigator and to their primary care provider by telephone without the use of a computer	Participants in the home tele-monitoring and community-based monitoring groups had clinically and statistically significant reductions in both systolic blood pressure and diastolic blood pressure during a 3-month monitoring period as compared with the participants in the usual care group.

Baer et al. [33]	Wounds were photographed by a homecare nurse using a digital camera, and the images were transmitted to a server from the nurse's office, together with patient details. The homecare nurse graded the wounds and suggested a treatment plan. Subsequently, a specialist wound care nurse also graded them and suggested a treatment plan, using the data stored on the web server, Home Telehealth Consultation System.	The results were encouraging and suggest that web-based communication can improve the quality of care for patients with leg wounds and can reduce costs.

Barnason et al. [34]	Effect of a home communication intervention (HCI) to augment home healthcare (HHC) on functioning and recovery outcomes of elderly patients undergoing coronary artery bypass graft. The experimental group in this study received HCI using a technology device called the Health Buddy. The Health Buddy device is a small, simple communication device, approximately 6 × 9 inches, with an illuminated screen and four large buttons for the patient to use to interact with messages viewed on the screen.	HCI subjects, compared with the HHC group only, had a significantly higher adjusted mean general health functioning score. There were significant time effects on physical, role-physical, and mental health functioning, indicating that both groups improved over time. The groups reported similar postoperative problems; however, the control group had more emergency department visits than the HCI group.

Barnason et al. [35]	The home communication intervention (HCI) was delivered to coronary artery bypass graft patients with ischemic heart failure, using a device called the Health Buddy. This small device attaches to the patient's telephone as a means of communication and provides healthcare professionals with assessment of patient symptoms (e.g., fatigue or sleep problems) and strategies to manage reported symptoms.	Findings demonstrate promise for the potential usefulness of a targeted intervention for a vulnerable subsample of coronary artery bypass graft patients during the early recovery period. Furthermore, the uniqueness of the telehealth device used provided clinicians with another potential option for maintaining contact with high-risk patients. Self-efficacy is a key component to self-care and disease management.

Benatar et al. [36]	Care was delivered by the homenurse visit or the nurse telemanagement method. In the latter, patients used trans-telephonic home monitoring devices to measure their weight, blood pressure, heart rate, and oxygen saturation. These data were transmitted daily to a secure Internet site. An advanced practice nurse worked collaboratively with a cardiologist and subsequently treated patients via telephone.	The results demonstrate significant improvements in outcomes and quality of care for patients with severe heart failure using aggressive remote tele-monitoring versus traditional homenurse visits. The data provide evidence that the introduction of current state-of-the-art computerized technologies allows rapid and accurate monitoring of patients with severe heart failure. The combination of these technologies and heart failure management by an advanced-practice nurse under the guidance of a cardiologist is cost effective and leads to improved outcomes and care.

Bendixen et al. [37]	The effects on healthcare costs of a Veterans Administration telerehabilitation programme were examined. LAMP (Low Activities of Daily Living (ADL) Monitoring Programme) is based on a rehabilitative model of care. LAMP patients received adaptive equipment and environmental modifications, which focused on self-care and safety within the home. LAMP care coordinators remotely monitored their patients' vital signs and provided education and self-management strategies for decreasing the effects of chronic illnesses and functional decline.	No significant differences were detected in postenrollment costs between LAMP and the matched comparison group. For LAMP patients, the provision of adaptive equipment and environmental modifications, plus intensive in-home monitoring, led to increases in clinic visits after intervention with decreases in hospital and nursing home stays.

Bohnenkamp et al. [38]	After discharge from the hospital, cancer patients with new ostomies were assigned to one of two groups: home health visits only or home health plus telenursing contact. The home health group received home health visitations by a nurse who continued evaluations and education according to current management protocols.	The telenursing group was more satisfied with care after discharge from the hospital and required fewer pouch changes, so care was less expensive because of the decreased number of pouches used. The telenursing patient group believed that the ostomy nurse understood their problems better than the home health nurse did, and they were more comfortable with information provided by the ostomy nurse. The telenursing group received care from nurse specialists who were able to individualize patient care, decrease cost, and improve patient satisfaction.

Bowles and Dansky [39]	Nurses in a large, urban homecare agency used televideo technology to improve the self-management of diabetes for older adults who were admitted for skilled home care.	Telehomecare is a new teaching and monitoring tool that helps patients improve their knowledge and self-management of diabetes. Telehomecare visits are effective for reinforcing patient education and achieve significant improvements in self-management. Patients in the video group received more contact with their nurses in person and via video visits versus in-person visits.

Bowles et al. [40]	Effects of evidence-based disease management guidelines were delivered to patients with heart failure and diabetes using three different modalities: in-person visits alone (control), in-person visits and a telephone intervention (telephone), and in-person visits and tele-monitoring (tele-monitoring). Three different kinds of telehealth monitors were used. Two monitors provided physiological monitoring, with a blood pressure cuff, bodyweight scale, glucometer, and pulse oximeter. The third monitor provided these in addition to a digital stethoscope and videoconferencing. The telehealth information was transmitted to the agency where it was monitored daily by the nurses. Nurses assessed physical and emotional status, reviewed medications, and instructed the patients on self-care and disease management.	There was no difference between the groups in the primary outcome (rehospitalization), although there was a trend toward increased hospital readmissions in the telephone patients versus control. Having heart failure and receiving more in-person visits were significantly related to readmission and time to readmission. However, the differences between the three groups were nonsignificant. There was a trend for increased risk of readmission for the telephone group and for readmission sooner. Patient rehospitalization and emergency department visit rates were lower than the national average, making it difficult to detect a difference between groups.

Brennan et al. [41]	Nursing practice capitalizes on a web-based resource (HeartCareII) to support patient self-management, symptom interpretation, and self-monitoring. Research staff provided computers and technical assistance; visiting nurses trained patients in the components of the HeartCareII website most relevant to their care needs.	The duration of visiting nurse association (VNA) service and use of HeartCareII resources vary across patients and nurses.

Buckley et al. [42]	Remote monitoring equipment and a video-phone operating over a standard telephone line were installed in the homes of patients with diabetes, and they were trained in their use and operation. Residents performed daily monitoring of blood pressure and/or blood glucose using the equipment. The residents received weekly video visits from the nurse educators.	The results demonstrated a trend in reduction of HbA1c for the residents with diabetes, but there was no significant improvement in HbA1c, blood glucose, or blood pressure measurement. Knowledge of diabetes and hypertension, self-efficacy, and perception of telehealth significantly increased following the protocol.

Buckley et al. [43]	The wound, ostomy, and continence (WOC) nurse first completed a wound assessment and recommendation form based on a verbal report from the homecare nurse then accessed digital images of the wounds and made any indicated modifications to the original assessment and management plan, providing a rationale for any changes. Comparisons were made between the assessment completed by the homecare nurse and the WOC nurse's assessment and between the WOC nurse's assessment and recommendations based only on a verbal report, and his or her assessment and recommendations based on the combination of a verbal report and a digital photograph.	There was a high percentage of agreement between the wound assessments completed by the homecare nurse and those completed by the WOC nurse; areas of disagreement often impacted the overall assessment. WOC nurses who provide remote nurse-to-nurse consultations without directly visualizing the patients' wounds through digital images are at risk for under- or overtreating patients' wounds. Digital images also provide an opportunity for the WOC nurse to mentor homecare nurses in wound assessment and care.

Buckley et al. [44]	The telehealth nurse scheduled a series of two initial home visits to stroke patients at home and follow-up weekly telehealth visits with each caregiver over a six-week period. The telehealth equipment was installed in the patient's home.	Major factors related to the receptiveness of telehealth were the timing of when it was offered after discharge and the level of caregiver burden. Caregivers expressed the opinion that the option of using telehealth should be introduced at the time of the stroke survivors' discharge, when they were trying to cope with new needs and responsibilities. The majority of caregivers who had elected to use the telehealth reported having a moderate level of patient dependence upon them and a low-to-moderate level of burden was consistent with the caregivers' comments of needing additional support offered by telehealth and of being moderately comfortable with and interested in technology.

Cardozo and Steinberg [45]	Recently discharged older patients received a nurse visit up to 3 times/week and home telemedicine monitoring on a daily basis. The telemedicine component used remote monitoring to assess the patient's health status. It had the capability to monitor respiratory rate, blood pressure, pulse oximetry, and patient weight and continually graph and update the electronic patient record. These data were available to the healthcare team and allowed them to improve care coordination and provide proactive and individualized management. It also included the Health Buddy appliance that provided important patient health education and self-regulating disease management information.	A majority of patients showed improved quality of health perception, better disease understanding, and high satisfaction rates with telemedicine. A home-based, case-managed telemedicine care system is cost-effective and improves health outcomes in older patients who are at risk from deteriorating health and further deconditioning as a consequence of repeated hospital admissions. Telemedicine is well accepted by the elderly as a complementary modality of care.

Chae et al. [46]	In home health services (HHS) for elderly patients, a telemedicine system with a 33-kbs narrow-band approach to determine the effectiveness in providing quality services was implemented and evaluated. A computer-based patient record was also developed to view a patient summary and to document encounters at the patient's home.	Telemedicine was effective in terms of reducing the number of clinic visits and achieving patient satisfaction; 72% of patients were satisfied with telemedicine, but patient location showed a significant difference for patient satisfaction. Patients in their homes were more satisfied than patients in nursing homes. Of four types of services provided, medical consultation was the most highly satisfactory service with telemedicine, followed by physical therapy. Although the satisfaction scores did not indicate a significant difference in the system characteristics, the quality of verbal communication appeared to be a more important factor in influencing patient satisfaction than set-up time or quality of image. This approach enabled a physician to accurately assess elderly patients in their homes or nursing homes and to treat them with the help of a home-visiting nurse.

Chambers and Connor [47]	An interactive software programme was designed to provide family caregivers with information, advice, and psychological support by way of feedback of their coping capacity. The multimedia programme consists of an information-based package that provides caregivers with advice on health promotion and relaxation and offers them a range of coping strategies (e.g., positive self-talk, assertiveness training, and relaxation tapes and videos). The programme also includes a caregiver's self-assessment instrument, designed to provide both family and professional caregivers with information to assess how family caregivers are coping with their caregiving roles.	The programme is useful to caregivers and of high quality and efficient in relation to utility and usability. The programme was highly rated in terms of global usability and its five component scales of attractiveness, controllability, efficiency, helpfulness, and learnability. This illustrates that the programme is visually pleasant, easily understood, responds quickly, and corresponds with user's expectations. Users felt there was room for improvement in the navigation of the programme.

Chambers and Connor [48]	The interactive application consisted of an information-based package that provided caregivers with advice on the promotion of psychological health, including relaxation and other coping strategies. The software application also included a caregiver self-assessment instrument, designed to provide both family and professional caregivers with information to assess how family caregivers were coping with their caregiving role.	The findings evidenced that the majority of users found the software to be usable and informative. Some areas were highlighted for improvement in the navigation of the software.

Chang et al. [49]	Telehealth and telephone communication technologies were used by nurse practitioners to provide individualized diabetes care management and to have similar effects on glycemic control.	The number of days of participation in the programme was greater for the telehealth group than the group receiving the telephonic intervention, but this difference was not statistically significant. Approximately 75% of the patients worked with nurse practitioners and had reached individualized glycemic goals at disenrollment. Among these patients, those receiving the telehealth intervention had a 3.1% reduction in HbA1c, and those receiving the telephone intervention had a 2.7% reduction in HbA1c, over a mean period of 204 days. After disenrollment, HbA1c increased slightly, suggesting that veterans need continuous individualized care, in addition to routine followup, to manage their diabetes.

Cleland et al. [50]	Patients with a recent admission for heart failure and left ventricular ejection fraction were assigned randomly to home tele-monitoring, nurse telephone support, or usual care. Home tele-monitoring consisted of twice-daily patient self-measurement of weight, blood pressure, heart rate, and rhythm with automated devices linked to a cardiology center. The nurse telephone support (NTS) consisted of specialist nurses who were available to patients by telephone. Primary care physicians delivered usual care.	During 240 days of followup, 19.5%, 15.9%, and 12.7% of days were lost as the result of death or hospitalization for usual care, nurse telephone support, and home tele-monitoring, respectively (no significant difference). The number of admissions and mortality were similar among patients randomly assigned to nurse telephone support or home tele-monitoring, but the mean duration of admissions was reduced by 6 days with home tele-monitoring. Patients randomly assigned to receive usual care had higher one-year mortality than patients assigned to receive nurse telephone support or home tele-monitoring. Further investigation and refinement of the application of home tele-monitoring are warranted because it may play a valuable role in the management of selected patients with heart failure. Although many patients were elderly, their acceptance and ability to cope with the home tele-monitoring technology were high. Few patients asked for the equipment to be removed or failed to comply with daily measurements. Good or very good satisfaction with home tele-monitoring was reported by 96% of patients. Improved access to care, either by nurses or by tele-monitoring, appeared to lead to an increase in patient contacts.

Clemensen et al. [51]	Video consultations in the home of the patient were introduced. The video consultation setup constitutes a new organisational way of working, described as “a new triangle” based on immediate interindividual cooperation and teamwork. In the triangle, competences were combined, which led to a more holistic treatment and a more active patient role.	Competences were combined, which led to a more holistic treatment and a more active patient role. A spreading of knowledge among all participants was seen, resulting in an upgrading of the competences of the visiting nurse especially. The introduction of a real-time, online link between hospital and home constitutes the basis for simultaneous communication between all participants, resulting in a “witnessing” situation potentially securing or even enhancing quality of treatment.

Dang et al. [52]	A programme called telephone-linked care for dementia was conducted. This programme offered access to resources, as in the REACH trial and also provided caregiver education and periodic monitoring questionnaires using a screen-phone. The intervention was delivered via a CTIS screenphone. The system allowed users to make and receive calls and messages.	The respondents were more satisfied with the care coordination aspect of the programme than the education or the monitoring. The project suggests that care coordination aided by screen-phones may be a useful model for caregiver support in a managed care setting.

Dansky and Vasey [53]	Patients with heart failure received the Health Buddy and used it for the duration of home health services. The Health Buddy was programmed to ask patients questions related to heart failure including symptoms, self-care practices, and medication compliance. During the formal episode of care, all patients received standard care.	Patients who continued using telehealth beyond the formal episode of care showed greater improvements in respiratory status and activities of daily living. None of the patients who used telehealth during this stage had any hospitalizations or emergency department events, while 28.3% of the control group patients required hospitalization and 26.1% had at least one emergency department visit. Telehealth patients were more likely to report that they measured their weights daily and more likely to report an increase in diuretic dose following sudden weight gain, ankle swelling, or shortness of breath.

Dansky et al. [54]	“Telehomecare” is a telephone-based communication system with medical peripherals that is used in the home setting. Patients use the medical devices to assess their health status and transmit the data to clinicians for review and action. Nurses and other clinicians use the data to monitor patients' health and teach patients and their caregivers self-management behaviours. Measurement and transmission of blood pressure, temperature, weight, blood glucose levels, and pulse oximetry are possible. The one-way systems are used independently by the patient and are typically programmed to be used every day at a predetermined time. If the nurse who checks the transmitted data observes abnormal values, he or she may call the patient or the homecare nurse for further information or intervention. The two-way system adds a video camera and digital stethoscope to the monitoring device, permitting two-way synchronous interaction between nurse and patient.	Patients in the telehomecare group had a lower probability of hospitalizations and emergency department visits than did patients in the control group. Differences were statistically significant at 60 days but not at 120 days. Results show a greater reduction in symptoms for patients using telehomecare compared to control patients. The technology enables frequent monitoring of clinical indices and permits the home health care nurse to detect changes in cardiac status and intervene when necessary.

Dansky et al. [55]	Telehealth, a clinical information system that transmits data over ordinary telephone lines, was used by individuals in their homes to communicate electronically with healthcare providers. This study investigated the influence of telehealth on self-management of heart failure in a sample of older adults.	Confidence is a predictor of self-management behaviors. Patients using a video-based telehealth system showed the greatest gain in confidence levels with time. Managers and policy makers responsible for creating and funding programmes that support the use of health-information technologies by older adults can benefit from these results.

Dansky et al. [56]	The home health agency used a telehomecare model as a complement to traditional home visits. The system connects a central station with patients' units over ordinary telephone lines using an internal modem. The central station combines a windows-based PC with a touch-tone telephone to deliver full-color video and telephone-quality audio.	Telehomecare is highly structured and moderately complex. Nurses begin with simple tasks and move to more complex activities. The patient and the family are clearly the focus of telehomecare intervention.

Darkins et al. [57]	The Veterans Health Administration introduced a national home telehealth programme, Care Coordination/Home Telehealth (CCHT). Its purpose was to coordinate the care of veteran patients with chronic conditions and avoid their unnecessary admission to long-term institutional care. After a patient is enrolled in the programme, the care coordinator selects the appropriate home telehealth technology, gives the required training to the patient and caregiver, reviews telehealth monitoring data, and provides active care or case management (including communication with the patient's physician).	Routine analysis of data obtained for quality and performance purposes shows the benefits of a 25% reduction in number of bed days of care, a 19% reduction in the number of hospital admissions, and a mean satisfaction score rating of 86% after enrollment in the programme. The cost of Care Coordination/Home Telehealth is less than the other noninstitutional care programmes and nursing-home care. The Veterans Health Administration experience is that an enterprise-wide home telehealth implementation is an appropriate and cost-effective way of managing chronic-care patients in both urban and rural settings.

de Lusignan et al. [58]	The use of the programme allowed monitoring of vital signs, such as pulse, blood pressure, and weight, of patients with chronic heart failure. Data was then transferred to a tele-monitoring server at a hospital and could be viewed by clinicians. The telemedicine group has the ability to video consult. A comparison was made with a control group (traditional care).	Compliance with measuring weight, pulse, and BP remained high throughout the study. The data collection system and secure web server were reliable. The tele-monitoring group complied better with collecting prescriptions for their cardiac drugs. Video-consulting started with enthusiasm but became less useful. There were no significant differences in the quality of life and Chronic Heart Failure Questionnaire scores between the tele-monitored group and the controls.

DelliFraine et al. [59]	The relationship between telemedicine knowledge management activities and nurses' perceived efficiency and effectiveness of telemedicine in home health was investigated. Knowledge management enhances the processes of care for a variety of services in different settings, with varying degrees of usage by clinical staff. These knowledge management activities are intended to facilitate communication and information exchange between physicians, nurses, and patients, which in turn enhances patient care delivery.	Results indicate a significant association between combined explicit and tacit knowledge management activities using telemedicine and perceived efficiency and effectiveness of telemedicine. Telemedicine knowledge management activities might have a positive impact on perceived efficiency and effectiveness of care in home health.

Demiris et al. [60]	Videoconferencing and Internet equipment were used to enable interactions between patients and nurses. An instrument that measures perceptions of telehomecare was used.	There was no statistically significant change of perception in the control group. The experimental group showed an overall, more positive perception of the system, and the mean score difference was higher compared to the control group. Elderly patients evaluated their telehomecare experience as being positive, and they felt more comfortable with the technology, believing that the nurse can understand their medical problems over the television. The study suggested that patients tend to become more familiar with and confident in technology after participation in a telehomecare system, and the subjects seemed less concerned about telehomecare violating their privacy. The initial fears of some patients, like privacy, seemed to diminish. Some other original perceptions of telehomecare did not hold after exposure to the system. Patients' overall impressions of a telehomecare system were more positive after they had experienced it. They evaluated this experience as positive and beneficial for their own health as well as time saving for the nurses. They felt that a nurse could get a good understanding of their medical problems over the television and, therefore, accepted the underlying concept of telehomecare.

Elliott et al. [61]	To examine the effectiveness of an individualized problem-solving intervention delivered in videoconferencing sessions with family caregivers of persons living with a spinal cord injury and possible contagion effects on care recipients. Family caregivers were randomly assigned to an education-only control group or an intervention group in which participants received problem-solving training in monthly videoconference sessions for a year.	Older caregivers were more likely than younger caregivers to remain in the study. Intent-to-treat analyses projected a significant decrease in depression among caregivers receiving problem-solving training; efficacy analyses indicated this effect was pronounced at the sixth-month assessment. Care recipients of caregivers receiving problem-solving training reported gains in social functioning over time. Community-based, telehealth interventions may benefit family caregivers and their care recipients, but the mechanisms of these effects are unclear.

Finkelstein et al. [9]	The study demonstrates that telehomecare linking homebound patients with their home healthcare nurses over a standard telephone system provides high-quality, clinically useful, and patient satisfactory interactions. Virtual visits, consisting of two-way audio and video interactions between the central site, home health care nurses, and subjects at home were compared for technical quality and clinical usefulness by the home health care nurses who performed the virtual visits.	All subjects were satisfied with their home health care; satisfaction increased with an increasing level of telehomecare intervention. Subjects receiving physiological monitoring and videoconferencing/Internet access in addition to standard care were most satisfied with their care. Virtual visits can be conducted over ordinary telephone systems. Patients can use telehomecare with moderate levels of training. These programmes can provide timely and quality home health nursing care with virtual visits augmenting traditional home visits.

Finkelstein et al. [62]	Patient outcomes and cost were compared when home healthcare was delivered by telemedicine or by traditional means for patients receiving skilled nursing care at home. A randomized controlled trial was established using three groups. The first group received traditional, skilled nursing care at home. The second group, the video intervention group, received traditional, skilled nursing care at home and virtual visits using videoconferencing technology. The third group, the monitoring intervention group, received traditional, skilled nursing care at home; virtual visits using videoconferencing technology; and physiologic monitoring for their underlying chronic condition.	Virtual visits between a skilled home health care nurse and chronically ill patients at home can improve patient outcomes at lower costs than traditional, skilled face-to-face home healthcare visits. Subjects who were both monitored and used videoconferencing had a better ADL rating at discharge than did the control group.

Forbat et al. [63]	An intervention with utility of a handheld side-effect monitoring system for people receiving chemotherapy in the homecare setting.	People affected by cancer were reflecting on issues such as power and surveillance in cancer care. While these terms are ordinarily considered to reflect negative elements of care, they were used by participants in an empowering manner. Patients receiving cancer care at home reported positive perspectives on the use of healthcare technology, thereby subverting the idea of surveillance as negative. Use of health surveillance technologies, which enable people to remain in their own homes during treatment, are likely to be well received.

Gray et al. [64]	An Internet-based telemedicine programme, Baby CareLink, was designed to reduce the costs of care and to provide enhanced medical, informational, and emotional support to families of very low-birthweight infants during and after their neonatal intensive care unit stay. Baby CareLink is a multifaceted telemedicine programme that incorporates videoconferencing and World Wide Web (WWW) technologies to enhance interactions among families, staff, and community providers.	Families in the CareLink group reported higher overall quality of care and significantly fewer problems with the overall quality of care received by their family. They also reported greater satisfaction with the unit's physical environment and visitation policies. The frequency of family visits, telephone calls to the neonatal intensive care unit, and holding of the infant did not differ between groups. The duration of hospitalization until ultimate discharge to the home was similar in the two groups. All infants in the CareLink group were discharged directly to home whereas 20% of control infants were transferred to community hospitals before ultimate discharge home.

Guilfoyle et al. [65]	A protocol for the use of videophones in community health was developed. Clients with a range of health needs were equipped with a commercially available video-phone connected using the client's home telephone line. A hands-free speakerphone and a miniature video camera (for close-up views) were connected to the video-phone.	Both clients and nurses rated the equipment as satisfactory or better. None of the nurses felt that the equipment was difficult to use, including unpacking it and setting it up; only one client found it difficult. Taking into account the clients' responses, including their free-text comments, a judgement was made as to whether the video-phone had been useful to their nursing care. In seven cases, it was felt to be unhelpful, and in three cases, it was judged helpful.

Hauber and Jones [66]	Telerehabilitation was used to support families caring at home for individuals with prolonged states of reduced consciousness. Patients were discharged home with family members as the primary caregivers. Their families were followed for 4 to 8 weeks via video-phone. Follow-up telephone surveys were conducted with a family member 6 to 9 months after discharge and compared to surveys of a similar group that had not received the video-phone followup.	More patients in the videoconferencing group were still living at home and had returned for rehabilitation. Families in the video-phone group reported more of their needs met than families in the comparison group. The use of videoconferencing to bridge the transition to home for families caring for a family member may assist families in successfully caring for the individual in the home and reducing the number of perceived family needs.

Hirakawa et al. [67]	The aim was to clarify the possible changes brought about by the introduction of the long-term care insurance system in terms of number of communication/recording tasks, related nursing services in use, and when and where these tasks were performed. It was also to explore the advantages of introducing information technology (IT) systems into nursing service settings. The study was designed as a before-and-after study in two sessions, namely, before and after introduction of a long-term care insurance system. Different measurements were performed during the intervention.	Following the adoption of the new system, these tasks tended to occur mostly around the starting time of services. As for the staff, the involvement of the professional caregivers increased. Regarding content of communication/recording, reports, confirmation, and instruction increased.

Hofmann-Wellenhof et al. [68]	The feasibility and acceptance of teledermatology for wound management of patients with chronic leg ulcers by homecare nurses were examined. Patients with chronic leg ulcers of different origin were included. In initial in-person visits, leg ulcers were assessed and classified and underlying diseases noted. Follow-up visits were done by homecare nurses. Once a week, digital images of the wound and surrounding skin and relevant clinical information were transmitted via a secure website to an expert at the wound care centre. The experts provided an assessment of wound status and therapeutic recommendations.	In 89% of the 492 tele-consultations, the quality of images was sufficient or excellent, and experts were confident about giving therapeutic recommendations. Treatment modalities were changed or adapted in one-third of the consultations. There was a significant decrease in visits to a general physician or the wound care centre. The acceptance of teledermatology was high in patients, homecare nurses, and wound experts.

Horton [69]	Telecare service was given to patients living at home with chronic obstructive pulmonary disease (COPD) by a home care team using telecare service. Telecare service comprised the following elements: daily monitoring of the patient's condition and monitoring to investigate and determine any physiological changes via parameters as oxygen saturation, pulse, and respiratory rate.	The experience and expectation in telecare, the usability of equipment, and changes in practice can impact COPD care. The outcome highlighted that the rapid access to care, an increased sense of personal safety and security, and the continuity of care are perceived as benefits. However, the equipment was perceived as bulky and not user friendly.

Huddleston and Kobb [70]	Older veterans with chronic diseases and high healthcare utilization were followed with an in-home technology device, that is, the Health Buddy, and risk management software. Programme staff could identify at-risk patients based on their responses to a series of questions about symptoms, behavior, and knowledge. Patients were followed in the programme for at least six months.	The outcome showed a 45% decrease in hospital admissions, a 67% decrease in nursing-home admissions, a 54% decrease in emergency department visits, and a 38% decrease in pharmacy prescriptions. The patients also demonstrated improved compliance with treatment regimens, and both patients and providers reported high levels of programme satisfaction.

Jenkins and McSweeney [71]	A comparison among the effectiveness of three hospital discharge care models for reducing congestive heart failure–related readmission charges. The care models— home telecare delivered via 2-way videoconference devices with integrated stethoscope, nurse telephone calls, and usual outpatient care—were compared.	The outcome showed that the between-group difference was not statistically significant and cannot offer incremental benefits beyond telephone followup; it is also more expensive.

Jerant et al. [72]	The trial compared 3 posthospitalization nursing-care models for reducing congestive heart failure (CHF) readmission charges during 180 days of followup. Subjects received in-person visits at baseline and at 60 days, plus one of three care modalities in the interim: video-based home telecare, telephone calls, or usual care.	CHF-related readmission charges were more than 80% lower in the telenursing groups compared to usual care, and these groups also had significantly fewer CHF-related emergency visits. In-person visits were more than three times longer than telenursing visits (*P* < 0.0001), only partially due to added travel time. Patient self-care adherence, medications, health status, and satisfaction did not significantly differ between groups. Telenursing can reduce CHF hospitalizations and allow increased frequency of communication with patients.

Jerant et al. [73]	Homenurse visits after discharge can reduce readmissions for persons with congestive heart failure (CHF), but the intervention costs are high. To compare the effectiveness of three hospital discharge care models for reducing CHF-related readmission charges: (1) home telecare delivered via a two-way video-conference device with an integrated electronic stethoscope; (2) nurse telephone calls; and (3) usual outpatient care.	CHF-related readmission charges were 86% lower in the telecare group and 84% lower in the telephone group than in the usual care group. However, the between-group difference was not statistically significant. Both intervention groups had significantly fewer CHF-related emergency department visits and charges than the usual care group. Trends favouring both interventions were noted for all other utilization outcomes.

Kawaguchi et al. [74]	The Internet-based system allows patients (equipped with a laptop computer), nurses, and physicians to access information from a central database through a wireless network. E-mail and video mail messages as well as vital signs data can be sent daily by the patient to a server at a regional healthcare centre and can be accessed by a nurse or physician, who can then decide on appropriate care.	The system was tested by a male patient with type 2 diabetes mellitus to see whether it would enhance his own management of his condition. During a 71-day period, educational material was provided. The telenursing system helped the patient to manage his condition, as shown by significant improvements in his levels of blood glucose and glycosylated haemoglobin (HbA1c) and in blood pressure. Findings suggest that the system is feasible.

Kearney et al. [75]	The acceptability of using handheld computers as a symptom assessment and management tool for patients receiving chemotherapy for cancer was evaluated. The patients used the handheld computer to record and send daily symptom reports to the cancer centre and receive instant, tailored symptom management advice during two treatment cycles.	Patients believed the handheld computer had improved their symptom management and felt comfortable using it. The health professionals also found the handheld computer to be helpful in assessing and managing patients' symptoms. The hand-held, computer-based symptom management tool was feasible and acceptable to both patients and health professionals in complementing the care of patients receiving chemotherapy.

Keaton et al. [76]	Caregivers answered questions through the use of Caring-web, which is a web-based intervention for caregivers of people with stroke. The e-mail messages from caregivers were then answered by a nurse specialist and members of an e-rehabilitation team. (Caring-web enables to provide different types of education and support to assist caregivers' needs.)	The outcome showed that the caregivers' questions centered on medication management (19%), community and government service (23%), and stroke and related issues in dealing with stroke (58%). This indicated that the caregivers were seeking new knowledge so they could maintain themselves and their care recipients.

Kleinpell and Avitall [77]	The intervention consisted of in-hospital-based screening for discharge needs. A home telehealth monitoring system for transmission of weight, blood pressure, heart rate, and pulse oximetry was installed in the patient's home. Telephone followup was conducted when parametres were out of preset and for postdischarge followup on days 1 and 3 and weekly for 4 weeks.	Subjects were receptive to having the telehealth technology in the home and related positive experiences to having telephone followup to reinforce the discharge plan and to monitor postoperative recovery.

Kobza and Scheurich [78]	The utilization of telemedicine in situations where wound specialists consulted with the home health nurse in the patient's home regarding care of chronic wounds was examined. During the two-way video visit, the wound specialist assessed the patient and the wounds and made recommendations for treatment. The wound specialist also collected outcome data during the visits. This data was then compared with like data collected as a baseline prior to the telemedicine intervention.	Results revealed improved healing rates, decreased healing time, decreased number of home health visits, and a decreased number of hospitalizations related to wound complications. Telemedicine was deemed a viable option for delivering quality, cost-effective care to chronic-wound patients in the homecare setting.

LaFramboise et al. [79]	The feasibility of providing a heart failure disease management programme was studied through an in-home telehealth communication device (that is, Health Buddy). The effectiveness of the Health Buddy was compared with traditional home management strategies (telephonic, home visit) in achieving selected patient outcomes (self-efficacy, functional status, depression, and health-related quality of life).	Those who received telephonic disease management experienced decreased confidence in their ability to manage their heart failure, whereas all other groups experienced increased confidence. The results also indicated improvement over time with no group differences for functional status, depression, or health-related quality of life. These findings suggest that delivering a disease management programme through a telehealth communication device is feasible and may be as effective as traditional methods.

LaFramboise et al. [80]	Patients with heart failure used a Health Buddy for self-management. They were asked seven questions daily about heart failure symptom status and ability to follow the prescribed regimen.	Participants found that the Health Buddy is technically easy to use; that it promoted, taught, and supported heart failure self-management; and that it was even a “lifesaver,” but that it could be bothersome, complex, and a too lengthy intervention.

Larsen et al. [81]	Universal Mobile Telephone System (UMTS) mobile phones for video consultations in the home were tested. Patients with diabetic foot ulcers were offered three video-consultations instead of visits to the hospital outpatient clinic. The consultations took from 5 to 18 minutes. In all consultations, the hospital experts were able to assess the ulcer in cooperation with the visiting nurse and to decide on treatment.	Technical problems sometimes made it difficult. Even connectivity problems occurred in about half of the cases. In addition, the audio signal was rather unstable at times. In all situations except one, the clinicians were able to reach a decision that the expert felt confident about. After all consultations, the atmosphere and participants' attitudes were very positive.

Lillibridge and Hanna [82]	A telehealth technology was used to assist case managers to effectively manage their caseloads of HIV/AIDs clients, increase responsiveness to clients' changing medical conditions, and serve as a partial solution to the ongoing nursing shortage. Telehealth monitors were placed and used in the clients' homes for a period of four months.	The findings suggest that the use of telehealth technology has the potential to effectively assist case management and home health agencies, manage their caseloads, increase responsiveness to a client's changing medical conditions, and address the ongoing nursing shortage.

Lin and Yang [83]	Asthma care mobile service (ACMS) was performed in the carrying out of the intervention. ACMS is a care platform for asthma patients that uses mobile phones to monitor asthma patients' real-time conditions. The patient's breathing, coughing extent, sleep quality, and daily routine circumstances were recorded using the mobile phone, and the data were sent to NCHC's network platform. General practitioners could detect the location of the patient and, in real time, obtain information on the local climate and air quality. NCHC analysed and recorded the information. Physicians could evaluate whether or not there was a disease crisis on the basis of data changes. If an asthma event occurred, it was possible to inform the patient to come to the hospital by using the same communication system. The health education center provided medical information to patients so they could better understand changes in their diseases and their doctors' recommendations.	The results indicated that the most critical factor affecting behavioral intentions related to ACMS is user attitude, followed by perceived usefulness, subjective norm, perceived ease of use, and innovativeness. The results provide governments developing high-tech, preventive medicine strategies with the necessary data to define an appropriate policy to use in attracting greater participation in the effort.

Lindberg et al. [84]	The experience of certified paediatric nurses (CPNs) with the use of videoconferencing between the neonatal intensive care unit and the families' homes has been studied. Families were given a home videoconferencing unit, which allowed them to have contact and communicate with staff at the neonatal unit day and night.	The results showed that the nurses found that videoconferencing helped them to assess the overall situation at home and facilitated the relationship between parents and the infant. The CPNs felt that they were able to provide security to the family. The use of videoconferencing was considered to be a generally positive experience and a tool to improve nursing care at home.

Lindberg et al. [85]	Parents of preterm infants used real-time videoconferencing between their home and the neonatal intensive care unit (NICU) as a support after taking their infant home. Via video and sound in real-time, parents had access, day and night, to NICU staff.	The results showed that security provided access to the staff and face-to-face supportive meetings. Parents experienced videoconferencing as positive, which empowered them and gave them confidence in their new situation of being at home with their infant.

Lindberg et al. [86]	Videoconferencing was used between midwives and parents at home in order to support parents who were discharged early after childbirth.	The main reasons for contact were routine and the most frequent advice concerned breastfeeding. The quality of sound and picture was judged to be good and very good. The results showed that the meetings with videoconferencing were easy to handle and useful for making assessments and were a valuable and functional complement to usual practice, almost like a real-life encounter. The results suggest that videoconferencing may be a useful tool in postpartum care.

Lutz et al. [87]	The feasibility of using a hometelehealth system for assessing stroke patients' physical functions, depression, fear of falling, and their family caregivers' burdens was examined. A hometelehealth programme that was a stroke-specific, care coordination, hometelehealth (CCHT) programme was used. Data were transmitted via home telephone lines, which interfaced with a web-based programme that connected with registered nurses who reviewed the data and recorded information in the computerized patient record system.	The outcome indicated tailoring CCHT to individual needs. The patients believed the home health programme was beneficial and served as an important safety net and assurance during the initial period of returning home after discharge. The results provide opportunities for tailoring the programme's implementation.

Lutz et al. [88]	The purpose was to identify postdischarge needs of stroke patients; their caregivers described their experiences of using a care-coordination hometelehealth (CC/HT) programme to address their needs.	All study participants believed that a hometelehealth programme could be beneficial to their stroke recovery at home, and that it provided a safety net and a sense of security that a healthcare professional was monitoring their health. The findings suggest that a comprehensive care-coordination programme that includes hometelehealth could aid veterans and their caregivers in managing stroke recovery across the continuum of care at home and within the community.

Mair et al. [89]	An ethnographic study embedded in an RCT of home- telecare for people suffering acute exacerbation of chronic obstructive pulmonary disease (COPD) was conducted. Participants were randomized to receive either face-to-face home nursing support or a home- telecare support service. The telecare service consisted of a video-phone link and attachments that permitted remote physiological monitoring of blood pressure, pulse, temperature, and pulse oximetry. Both specialist respiratory nurses and patients took part in the trial and reported their experiences.	The telecare service did not provide an interactional advantage for the nurses providing this service and did not fit with the nurses' views of the most appropriate or preferred use of their skills. The telecare service seemed unlikely to become normalized as part of routine healthcare delivery because the nursing team lacked confidence that it was a safe way to provide healthcare in this context, and it was not perceived as improving efficiency.

Marineau [90]	People with acute infections transitioning in the home with support by an advance practice nurse used a telehealth system with advanced practice nurses (APNs) as a support when they were acutely ill. APNs used equipment to assess the physiological and psychological status of individuals transitioning from an acute infection in their home. This care included interventions conducted by the APN via telehealth, which mimicked all the essential components that would be accomplished in the hospital with the exception of being able to physically touch the participant.	The transition that occurred when an individual with an acute infection was discharged from the hospital to the home supported by telehealth technology revealed an overall positive experience. The findings highlighted the importance of the participants having a sense of control when recovering from their illnesses, which could be achieved at home with a family member acting as a substitute nurse. The participants shared that the hospital environment may not be optimal for recovering from an illness.

McCall et al. [91]	The feasibility of using mobile phone-based technology (that is, Advanced Symptom Management System in Palliative Care (ASyMSp)) was tested to monitor and manage symptoms reported by patients being cared for at home in the advanced stages of their illness and was carried out in two rural communities.	The system was usable and acceptable to patients and the health professionals who cared for them.

McCann et al. [92]	A mobile phone-based advanced symptom management system (ASyMS) on chemotherapy-related toxicity in patients with lung, breast, or colorectal cancer was evaluated. Patients used the mobile phone to record their symptoms, sending their reports directly to the nurses at their clinical site.	Patients reported many benefits of using ASyMS including improved communication with health professionals and improvements in the management of their symptoms. ASyMS has the potential to positively impact the management of symptoms in patients receiving chemotherapy treatment.

McGee and Gray [93]	A symptom management system was developed and implemented on personal digital assistants (PDAs) for use by cancer outpatients in their daily management of chemotherapy symptoms. The system allowed patients to record their symptoms at home and send these data to their cancer centre. Patients could view personalized self-care advice and more general medical information. In addition, cancer care nurses were alerted about significantly high symptom scores and could contact the patient by phone.	The patients felt the system was rewarding, valuable, educational, and interesting but should be treated cautiously. Patients expected that using the system would be more challenging than they in fact rated it after the trial. They rated it more educational and more rewarding than traditional meetings. The staff anticipated that the system would be useful for monitoring patients' symptoms. After the trial, most of the staff suggested that the system had improved communication between them and the patients, and that the patients had immediate access to and contact with the hospital.

Miller et al. [94]	The intervention was delivered by way of a device called the Health Buddy to patients who had undergone coronary artery bypass graft (CABG) with diabetes, which delivers “daily sessions” or script and was used for six weeks with assessment of symptoms such as fatigue and pain.	No statistical differences between the intervention and the control groups were found. Improvements in psychosocial functioning were comparable between the two groups.

Moreno et al. [95]	The impacts on Medicare costs of providing a particular type of home telemedicine to eligible Medicare beneficiaries with type 2 diabetes were estimated. Two cohorts of beneficiaries living in two medically underserved areas were randomized to intensive nurse case management via televisits or usual care.	Informatics for Diabetes Education and Telemedicine (IDEATel) did not reduce Medicare costs at either site. Total costs were higher for the treatment group than for the control group. Although the telehealth system had modest effects on clinical outcomes (reported elsewhere), it did not reduce Medicare use or costs for health services.

Mullan et al. [96]	An electronic diary for home monitoring by lung transplant candidates to improve communication between candidates and the transplant team was used. Candidates were randomized into control (following standard telephone-reporting procedures) and intervention (using an electronic diary to record and transmit a range of health-related measures) groups.	Subjects used the diary without difficulty and with good compliance and were positive regarding contact based on diary use. There were no significant differences in clinical outcomes between groups. Changing diary questions might improve the effectiveness of electronic monitoring for lung transplant candidates.

Myers et al. [97]	Impact of home-based monitoring on the care of patients with congestive heart failure was examined. Home-based tele-monitoring as a therapeutic tool was used. The effectiveness of home tele-monitoring in patients recently discharged from the hospital was assessed. Patients were provided home tele-monitoring for a two-month period following hospital discharge. Home visit frequency, patient rehospitalization rate, emergency department use, quality of life, and healthcare costs were compared to those a similar usual care. Patients in the tele-monitor group transmitted their weight, blood pressure, and oxygen saturation daily to a tele-monitor nurse, who evaluated each patient with a follow-up telephone call.	Daily homecare tele-monitoring reduced the frequency of home-nursing visits, provided cost savings, and was associated with improved self-perceived quality of life.

Nilsson et al. [98]	District nurses' (DN) experiences of using information and communication technology (ICT) to communicate with chronically ill people in their homes were described. An electronic messaging programme via computers and mobile phones with an Internet connection was used, enabling DNs and the ill people to exchange messages to and from anywhere. The programme comprised different virtual rooms, and communication was via text messages.	The DNs felt that the technology increased accessibility to nursing care through a more direct communication with the ill person, meaning that a more trusting relationship could be created. The DNs also experienced that the use of ICT saved working time. This study indicates that the use of ICT for communication allowed the DN to better support a chronically ill person at home, leading to improved home nursing care. This method of communication cannot replace physical presence but can be seen as a complement to nursing care at home.

Nilsson et al. [10]	People with serious chronic illnesses who used information and communication technology (ICT) to communicate with their district nurse were studied. The intervention was performed using an electronic messaging programme. The programme was accessible to any computer with an Internet connection. The programme consisted of different virtual rooms, where people could communicate using text messages.	The participants' communication with the district nurse was improved because of easy accessibility and because they felt increased security. They felt there were fewer limitations and that their everyday life was improved, which can also be seen as an improvement in care.

Pangarakis et al. [99]	Lung transplant recipients used a telemedicine device, that is, an electronic home spirometer, to gauge the function of their lungs when they were away from the hospital or clinic setting. Healthcare providers review transmitted spirometry tests and user's symptom responses to detect early signs of infection and or rejection. Current home spirometry users have questions, concerns, and preferences about spirometry that may influence their daily adherence. The spirometer had the capability to deliver feedback messages to potentially address these questions and concerns.	Findings revealed categories for feedback messaging content such as education (general, lifestyle, and infection), goals, timing, technique, monitoring, and reminders (time sensitive, positive). Messages were created according to length, feasibility, past experience, and neutrality for electronic implementation. It is believed that pertinent automated electronic feedback messages will enhance home spirometry connection, raise confidence in spirometry usage, and influence daily adherence to the spirometry protocol. The content additionally serves as a foundation for establishing a plan of care individualized to each home spirometry user.

Phillips et al. [100]	Telehealth interventions were designed to reduce the incidence of secondary conditions among people with mobility impairment resulting from spinal cord injury. Patients received a video-based intervention for nine weeks, a telephone-based intervention for nine weeks, or standard follow-up care. Participants were followed for at least one year to monitor days of hospitalization, depressive symptoms, and health-related quality of life.	Health-related quality of life was measured using the Quality of Well-Being (QWB) scale. QWB scores did not differ significantly between the three intervention groups at the end of the intervention period. At year one after discharge, scores for those completing one year of enrollment were significantly higher for the intervention groups compared to standard care. Mean annual hospital days were 3.00 for the video group, 5.22 for the telephone group, and 7.95 for the standard care group.

Pierce et al. [101]	A site on the World Wide Web, called Caring˜Web, for online education and support for caregivers of individuals with stroke was developed to provide web-based, in-home support and education for caregivers of persons with stroke during the first year after hospitalization.	The educational needs of survivors of stroke and their caregivers were identified and information that these individuals sought was developed into an educational Tip of the Month component for Caring˜Web. The top 12 topics reported were used to create educational Tips of the Month on Caring˜Web.

Procter and Single [102]	Remote devices for daily home-monitoring of vital signs of patients living with multiple complex conditions were implemented. The equipment was installed in the patient's home and programmed to prompt the patient to undertake these observations on a daily basis at an agreed-upon time. Results were downloaded to a central web-based server, which was accessed daily by the project nurse.	The service reduced overall hospital admissions during the intervention compared to those before the intervention. The patients' and caregivers' confidence in managing diseases was increased. Home monitoring helped patients to communicate more effectively with the primary care team, which was thereby enabled to provide more effective responses to patients.

Przybylski et al. [103]	Patients with implantable cardioverter defibrillators were provided with remote monitoring to increase their safety by early detection of technical or medical malfunctions and to decrease the number of follow-up visits. Medical and technical events were reported by the remote monitoring system as well as interruptions in monitoring longer than 14 days.	The remote-monitoring system reported medical events in 48% of patients. In total, 32 event reports were generated due to the detection of ventricular tachycardia, ventricular fibrillation, ineffective defibrillation with maximal energy, and supraventricular tachycardia. There were no reports on technical abnormalities of the implantable cardioverter-defibrillator system. The longest break was caused by the patient's stay abroad. The remaining interruptions were caused by travel, hospitalisations, and a temporary stay in a place without sufficient GSM coverage. During the follow-up period, there were no interruptions in monitoring caused by transmitter or implantable cardioverter-defibrillator failure. Remote monitoring of implantable cardioverter-defibrillator recipients does not present technical difficulties and enables early detection of serious events in patients.

Quinn [104]	Low-technology equipment was used to improve care for patients with heart failure enrolled in a home health agency. The nine-week intervention was targeted toward the home health nurses and included telephone and home visits, a teaching tool, digital scales, and a log/notebook filled out by the patients in the study.	The intervention outcomes included decreased patient rehospitalisation, decreased symptoms of heart failure, and increased quality of life; it also improved the organization of nursing care for patients with heart failure. The common symptoms of heart failure such as fatigue, shortness of breath, and sleep disturbances were validated. The emergent care visits and reduced nursing visits allow provision of the care for patients with heart failure in a more effective and efficient manner than usual care.

Reis et al. [105]	An interactive multimedia program is described that would assess the patient and family member's level of preparedness for specific caregiving functions for prostate cancer and provide tailored skill-building vignettes on caregiving techniques. This program is designed for a hybrid delivery utilizing both web-based resources and a CD-ROM.	Feedback from prostate patients and family members from a cancer center on perceived needs for caregiving training underscores the potential value of a computer-supported intervention for some patients and families. Implementation of the software, marketing, and distribution will be guided in part by recent e-health experiences that leave many health professionals appropriately skeptical about the utility of such products. The concept of providing electronic health communication for consumers, particularly in the area of prostate cancer caregiving, is clearly valid for numerous reasons.

Safran et al. [106]	Parental use of an Internet-based educational and emotional support system, Baby CareLink, in a regional NICU programme. Baby CareLink was installed in NICUs in four area hospitals. Parents were offered access from hospital terminals and from any other Internet access point. Data on use of the programme was collected by the computer system.	Medicaid families who accessed three or more Baby CareLink web pages per day took their infants home 17.5 days sooner than families who used Baby CareLink less often. Among non-Medicaid families, more-frequent users of Baby CareLink took their infants home 14.3 days sooner. Self-help tools for parents may free up nursing resources for families with greater needs.

Sandberg et al. [107]	Patients with diabetes were provided with a specially designed home telemedicine unit that allowed them to videoconference with nurse case managers (NCMs) and dietitians, upload blood glucose and blood pressure readings, and access educational materials and individualized data displays. Subjects and NCMs/dietitians participated in videoconferences every 4 to 6 weeks (with significant need, every 2 weeks) to educate patients, facilitate goal setting/self-management, and discuss concerns. Supportive interactions provided contact tailored to individual needs toward the goals of improved glycemic control, diabetes self-care, and other health outcomes.	Providers were very satisfied with their experience and felt their efforts with patients were generally successful. Providers also identified a number of unique benefits of telehealth interventions, such as opportunities for more frequent contact with patients, greater relaxation and information due to the ability to interact with the patients in their own homes, increased ability to reach the underserved, more timely and accurate medical monitoring, and improved management of data. The primary disadvantages identified were technology problems and a concern about the lack of physical contact with patients.

Scalvini et al. [108]	General practitioners received a portable electrocardiograph that transmitted a 12-lead ECG readout to a receiving station via a mobile or fixed telephone. ECG traces recorded were transferred, in real time, to receiving stations where cardiologists were available 24 hours a day for ECG referral and interactive tele-consultations. Patients in the home-based tele-monitoring group received a portable device that transferred by mobile or fixed telephone to a receiving station where a nurse was available for reporting and interactive tele-consultation. The patient could call the centre when needed (teleassistance) or the clinical team could call the patient for a scheduled appointment (tele-monitoring).	At the first telephone contact, a lower number of general practitioners' patients than the home-based tele-monitoring patients were on beta blocker, diuretic, and angiotensin-converting enzyme (ACE)-inhibitor therapy. The mean number of telephone calls was 2.6 per patient in the general practitioner group and 16.6 per patient in the home-based tele-monitoring group. This program, involving the patients directly, is able to reduce hospitalizations and decompensation episodes. The telecardiology service is able to solve the majority of GPs' questions, combining their knowledge of their patients, with the cardiologists' expertise in problems connected with CHF. In this case, telemedicine could be an opportunity for the GPs to follow their patients, contributing to improved management, therapy, and appropriateness of hospital admissions.

Scalvini et al. [109]	The feasibility of home-based tele-cardiology for patients with chronic heart failure (CHF) was assessed. CHF patients were enrolled into a programme of telephone followup and single-lead electrocardiography (ECG) monitoring. The patients transmitted their ECG data by fixed telephone line to a receiving station, where a nurse was available for an interactive tele-consultation.	A total of 124 cardiovascular events were recorded. Modifications to therapy were suggested in response to 119 calls; hospital admissions were suggested for 13 patients, further investigations for 7, and a consultation with the patient's general practitioner for 13. Twenty-two ECG abnormalities were recorded. In 63 patients receiving the beta-blocker carvedilol, the mean dosage increased from 36 to 42 mg. In the previous year, there were 1.8 hospitalizations per patient, while in the follow-up period there was 0.2 hospitalization per patient. Following up CHF patients using a nurse-led tele-cardiology programme seems to be feasible and useful.

Schwarz et al. [110]	The purpose was to examine whether tele-monitoring by an advanced-practice nurse reduced subsequent hospital readmissions, emergency department visits, costs, and risk of hospital readmission for patients with HF. Patient/caregiver dyads were randomized into two groups after discharge. Participants were interviewed soon after discharge and 3 months later about effects of tele-monitoring on depressive symptoms, quality of life, and caregiver mastery.	There were no significant differences related to tele-monitoring for any outcomes. Caregiver mastery, informal social support, and electronic home monitoring were not significant predictors of risk of hospital readmission. Further studies should address the interaction between the advanced-practice nurse and follow-up intervention with tele-monitoring of patients with HF to better target those who are most likely to benefit.

Sevean et al. [111]	Patients' and families' experiences with video telehealth consultations as a method of healthcare delivery in rural/remote communities were accessed.	Patients' and families' experiences of their telehealth visits were centered on three key themes: lessening the burdens (costs of travel, accommodations, lost wages, lost time, and physical limitations); maximizing supports (access to family, friends, familiar home environment, nurses, and other care providers); and tailoring specific e-health systems to enhance patient and family needs.

Shea et al. [112]	Participants in the intervention group received a home telemedicine unit (HTU) developed specifically for IDEATel (American Telecare, Inc., Eden Prairie, MN, USA). The HTU consisted of a web-enabled computer with modem connection to an existing telephone line. The HTU provided four major functions: videoconferencing over standard telephone service (POTS), allowing patients to interact with nurse case managers; remote monitoring of glucose and blood pressure with electronic upload and integration with dial-up Internet service-provider access to a web portal providing access to patients' own clinical data; secure web-based messaging with nurse case managers; and access to an educational website.	Telemedicine case management improved glycemic control, blood pressure levels, and total and LDL cholesterol levels at one year of followup.

Smith et al. [113]	The study determined the feasibility of using home audio/video telehealth equipment for administering nursing interventions to families, observing the client response, and collecting research data over specific intervals of time. The subjects were adult patients' nighttime mechanical ventilators for obstructive sleep apnea. Skin color, vital signs, spirometry, and pulse oximetry data collected simultaneously through telehealth equipment and through nurse observation in the home were the same.	Nursing interventions, equipment demonstrations, visual illustrations, and audiotaped educational directions were used to facilitate patient care; they were transmitted across telehealth with a few exceptions. Costs of telehealth visits were less than traditional home visits, and client evaluations of telehealth were positive.

Smith et al. [114]	Whether a telehealth intervention could improve compliance with continuous positive airway pressure (CPAP) by patients with sleep apnea was tested. These patients had been nonadherent to the initial three months of therapy, even after receiving the initial standard and then supplemental audiotaped/videotaped patient education for adhering to CPAP nightly. Interventions were delivered by nurses to two groups in their homes by telehealth over a 12-week period.	Both groups rated telehealth delivery positively. Telehealth interventions are a potentially cost-effective service for increasing adherence to prescribed medical treatments.

Stricklin et al. [115]	Patient response is a critical aspect of successful POC technology (point of care technology) implementation. The results of a pilot POC patient satisfaction study conducted at four home health agencies were presented.	Results support patient/caregiver satisfaction with POC technology use during the home visit. The top variables influencing patient receptiveness to the nurse's use of the computer in the home are those that closely relate to general satisfaction with homecare services. The patients want to be the nurse's first priority and focus; they do not want to be upstaged by the computer. Provided the computer does not create the perception of taking time or attention from the patient or inhibit verbal interaction, patients are likely to accept the nurse's use of a POC computer.

Tang et al. [116]	A pilot study on telepsychiatry was conducted. A videoconferencing link was established between a regional hospital and a care and attention home. Using this system, a psychogeriatric outreach team provided psychiatric assessments to residents of the care and attention home over 11 months.	Videoconferencing was found to be highly feasible. It was acceptable to staff and patients and more cost-effective than on-site visits.

Terry et al. [117]	The aim was to evaluate the effectiveness of telemedicine (TM) with digital cameras in treating wounds in a homecare setting. Subjects were randomly assigned to one of three groups.	Telemedicine is a useful communication tool in wound management but with limited power when randomization does not include wound size or type. Two important benchmarks were established for home care.

Torp et al. [118]	A pilot study of how information and communication technology (ICT) may contribute to health promotion among elderly spousal caregivers. The objective was to explore whether use of ICT by informal caregivers of frail elderly people living at home would enable them to gain more knowledge about chronic illness, caring, and coping; establish an informal support network; and reduce stress and related mental health problems. Potential participants were close relatives of an elderly person with a diagnosis of a chronic illness dwelling in the same household who wished to continue caring for their relative at home.	Results did not reveal any reduction in caregivers' stress or mental health problems. Caregivers reported extensive use of the ICT service, more social contacts, and increased support and less need for information about chronic illness and caring. Contact with and support from other caregivers with similar experiences were particularly valued by participants. The intervention enhanced contacts with family and friends outside the caregiver network. Thus, it can be seen that ICT has the potential to contribute to health promotion among elderly spousal caregivers.

Wakefield et al. [119]	A home-based intervention for heart failure was evaluated. Differences in nurse and patient communication profiles between two telehealth modes were compared: telephone and video-phone; longitudinal changes in communication, nurse perceptions, and patient satisfaction were evaluated.	Nurses were more likely to use open-ended questions, back-channel responses, friendly jokes, and checks for understanding on the telephone compared to video-phone. Compliments were given and partnership was more common on the video-phone. Patients were more likely to give lifestyle information and approval comments on the telephone, and more closed-ended questions on the video-phone were used. Nurses' perceptions of the interactions were not different between the telephone and video-phone, nor did their perceptions change significantly over the course of the intervention. There were no significant differences in patient satisfaction between the telephone and video-phone.

van den Berg et al. [120]	The GP (general practitioner) delegated routine home visits to qualified practice employees (registered nurses). Eligible patients were provided with telecare devices to monitor disease-related physiological values.	The GPs agreed that delegating tasks to a qualified practice assistant relieves them in their daily work.

Varis et al. [121]	A telemedicine system, that is, Doc@home, was evaluated to assist blood pressure treatment to reach better blood pressure control among hypertensive patients.	Blood pressure control was improved during the three-month followup. Patient-to-Doc@home compliance was good, but study physicians found the system time consuming in the beginning. The Doc@home telemedicine system showed a promising approach in hypertension treatment but needs some further development and trained staff to become a still more practical alternative.

Whitten et al. [122]	A telehospice project was conducted in urban and rural regions. Data from patients receiving tele-hospice services in their homes was collected. Nurses were the primary providers of tele-hospice services and initiated the majority of routine televisits.	Patients were extremely satisfied with tele-hospice and often expressed frustration that nurses did not use the tele-hospice equipment more frequently.

Whitten and Mickus [123]	A home telehealth program for patients with chronic obstructive pulmonary disease (COPD) and/or congestive heart failure (CHF) was evaluated. Patients diagnosed with COPD and/or CHF who were prescribed home healthcare services were randomly assigned to an experimental group where they received home health care through a combination of traditional face-to-face and telemedicine visits and a control group where only conventional home care was employed.	In regard to patient perceptions of home telecare, patients were satisfied with the technology and the way that care was delivered via this modality.

Willems et al. [124]	A nurse-led tele-monitoring intervention compared with regular care in asthma was evaluated. The control group received regular outpatient care, while the intervention group used an asthma monitor with modem at home, with an asthma nurse as the main caregiver. Clinical asthma symptoms and medical consumption were measured by using diaries. Asthma-specific quality of life was also measured.	Improvement in followup but no statistically significant difference between the groups was observed. A tele-monitoring programme on its own is not a guarantee of success. The patient's perceptions of asthma-specific quality of life (daily functioning) should be a key element in asthma tele-monitoring programmes.

J. M. Winters and J. M. Winters [125]	A variety of experienced healthcare practitioners performed functional assessments of stroke subjects using a collection of validated scales by varying approaches (face-to-face, low-bandwidth, and high-bandwidth videoconferencing) in a randomized order. In a second study, undergraduate nursing students performed similar performance measures and taught an unfamiliar individual how to programme and use an intravenous pump device, take a tympanic temperature, or draw up insulin in a syringe. In the third study, advanced-practice nursing students assessed vital signs and performed cardiopulmonary assessments on community-dwelling subjects using low-bandwidth and face-to-face approaches. Healthcare practitioners and students generally preferred high-bandwidth approaches over low-bandwidth alternatives when videoconferencing was used.	Most participants and practitioners were satisfied with the encounters, regardless of the level of technology used.

Visco et al. [126]	Telehealth Wound Care Program implemented at a hospital home health agency and a hospital was used where the wound care provided for one patient was included and described in the case study.	Many benefits of telehealth as an adjunct to usual therapy in wound care were noted.

Vitacca et al. [127]	The feasibility of telemedicine for home monitoring of patients with chronic respiratory failure (CRF) discharged from hospital was assessed. The patients transmitted pulsed arterial saturation (pSat) data via a telephone modem to a receiving station where a nurse was available for a teleconsultation. A respiratory physician was also available. Scheduled and ad hoc appointments were conducted.	The home monitoring was feasible and useful for titration of oxygen, mechanical ventilation setting, and stabilization of relapses.

Vitacca et al. [128]	The aim was primarily to evaluate reduction in hospitalisations and, secondly, exacerbations, general practitioner (GP) calls, and related cost effectiveness of teleassistance (TA) for patients with chronic respiratory failure. Patients were randomised to two groups: an intervention group entered a one-year TA programme while controls received traditional care.	The TA group experienced significantly fewer hospitalisations, urgent GP visits, and acute exacerbations. COPD patients, as a separate group, had fewer hospitalisations, emergency room admissions, urgent GP calls, or exacerbations. After deduction of TA costs, the average overall cost for each patient was less than that for usual care. In chronic respiratory failure patients on oxygen or home mechanical ventilation, nurse-centred teleassistance prevents hospitalisations, while it is cost-effective. The chronic obstructive pulmonary disease group seems to have a greater advantage from teleassistance.

Vitacca et al. [129]	The use of telemedicine in support of weaning from invasive mechanical ventilation on a woman at home by means of a telepneumology programme (TPP) is described. Under telephone assistance of a pulmonologist and a TPP nurse tutor, the pulsed arterial saturimetric (pSaT), heart rate (HR), and breathing pattern tracing monitoring were transmitted via a home telephone line and the aid of the caregiver.	Many patients at home on ventilators could possibly be weaned through the use of remote monitoring and call center response, with only family/caregivers on-site.

Woodend et al. [130]	The impact of three months of telehome monitoring on hospital readmission, quality of life, and functional status in patients with heart failure or angina was tested. The intervention consisted of videoconferencing and phone line transmission of weight, blood pressure, and electrocardiograms	Tele-home monitoring significantly reduced the number of hospital readmissions and days spent in the hospital for patients with angina and improved quality of life and functional status in patients with heart failure or angina. Patients found the technology easy to use and were satisfied. Telehealth technologies are a viable means of providing home monitoring to patients with heart disease at high risk of hospital readmission to improve their self-care abilities.

Wälivaara et al. [131]	District nurses (DNs) from four healthcare centres had access to different kinds of distance-spanning technology with mobile devices and used it in their health care at home.	The results fall into 2 categories: the well-known technology at hospitals is new at home; the new technology opens up possibilities, but it also has limitations. The participants viewed the technology at home as something good and as something that could open up possibilities. At the same time, they placed the use of the technology in the hands of the staff, which indicates some degree of dissociation from the technology. The importance of personal meetings between patient and caregiver was very clearly stressed even when distance meetings could be performed and accepted. The participants expressed immense trust in the nursing staff and considered them responsible for the new technology at home.

Young et al. [132]	The effectiveness of telephone and video-phone followup for children and families after a child's scoliosis surgery was evaluated. At discharge, those in the intervention group were provided with a video-phone operating on the ordinary telephone network (PSTN).	Video-phone and telephone use provided care continuity for patients and their families following a child's back surgery. The relative effect of the video-phone and telephone technology depended on the fit between the characteristics of the patients and families and the capacities of the technology. When implementing telehealth for follow-up care, a participatory process is recommended to ensure a proper fit between user characteristics and technology.
